# Seeing the PDB

**DOI:** 10.1016/j.jbc.2021.100742

**Published:** 2021-05-04

**Authors:** Jane S. Richardson, David C. Richardson, David S. Goodsell

**Affiliations:** 1 Department of Biochemistry, Duke University, Durham, North Carolina, USA; 2 Department of Integrative and Computational Biology, The Scripps Research Institute, La Jolla, California, USA; 3 Research Collaboratory for Structural Bioinformatics Protein Data Bank, Rutgers, the State University of New Jersey, Piscataway, New Jersey, USA

**Keywords:** protein structure, protein folding, RNA structure, structural biology, X-ray crystallography, molecular graphics, ribbon drawings, all-atom contacts, visualization, science education and outreach, 1sns, 1LpL, Four-character codes starting with a number are accession codes at the PDB archive;we use lower-case except for L, to avoid ambiguity in any font, 3D, three-dimensional, AED, Advanced Electronic Design, Cα, Alpha carbon atoms in a protein chain, CaBLAM, Cα-Based Low-resolution Annotation Method that uses peptide CO orientations to diagnose incorrect backbone conformations even if Ramachandran φ,ψ values are restrained, Frodo, widely used, early model-to-map graphics on laboratory-accessible hardware, GRIP-75, the first model-to-map molecular graphics system, at UNC Chapel Hill, H, hydrogen atom (as in H-bond), Kinemage, a file using Dave Richardson's format for interactive molecular graphics, KiNG, Kinemage Next Generation, in Java, by Ian Davis and Vincent Chen, Mage, Dave's original program, in C, to display kinemage graphics, NOE, Nuclear Overhauser effect measurement of atom–atom distance, by NMR, ORTEP, Oak Ridge Thermal Ellipsoid Plot, a small-molecule line graphics system for drawing the ellipsoids of anisotropic temperature factors at each atom, PDB, Protein Data Bank, for experimental structures of macromolecules, PS300, or MPS, Evans & Sutherland calligraphic (vector-drawn) display workstation, RCSB, Research Collaboratory for Structural Biology;US branch of the wwPDB, RDC, Residual dipolar coupling measurement of atom–atom orientation, by NMR, SOD, Cu,Zn superoxide dismutase, SS bond, disulfide bond, Suite, the sugar-to-sugar, rather than nucleotide, parsing of RNA backbone (the Richardsons' best published pun), TIM, triose phosphate isomerase, vdW, van der Waals, wwPDB, worldwide PDB, including RCSB, PDBe, PDBj, BMRB, and EMDB

## Abstract

Ever since the first structures of proteins were determined in the 1960s, structural biologists have required methods to visualize biomolecular structures, both as an essential tool for their research and also to promote 3D comprehension of structural results by a wide audience of researchers, students, and the general public. In this review to celebrate the 50th anniversary of the Protein Data Bank, we present our own experiences in developing and applying methods of visualization and analysis to the ever-expanding archive of protein and nucleic acid structures in the worldwide Protein Data Bank. Across that timespan, Jane and David Richardson have concentrated on the organization inside and between the macromolecules, with ribbons to show the overall backbone “fold” and contact dots to show how the all-atom details fit together locally. David Goodsell has explored surface-based representations to present and explore biological subjects that range from molecules to cells. This review concludes with some ideas about the current challenges being addressed by the field of biomolecular visualization.

Across most of the history of the PDB, we have shared a common passion for understanding and communicating the beauty and functional complexity of macromolecular 3D structures. Our approaches to this endless and satisfying challenge differ in a fundamental way, but because of that are highly complementary. Goodsell is fascinated with creating intuitive representations of what a single protein or a set of interacting molecules would look like, at a range of size scales, if it were possible to see their surfaces directly. The Richardsons are fascinated with how to show the internal organization—the connected backbone “fold” in 3D and the specific atom–atom contacts that determine the anatomy and individuality of the molecule, or the amazing specificity of interaction with another molecule. To celebrate the 50th anniversary of the Protein Data Bank (PDB) archive, we will give personal tours of our own experiences in seeing the PDB.

## The Richardson perspective

### Hand-drawn ribbons

**Figure 1:**
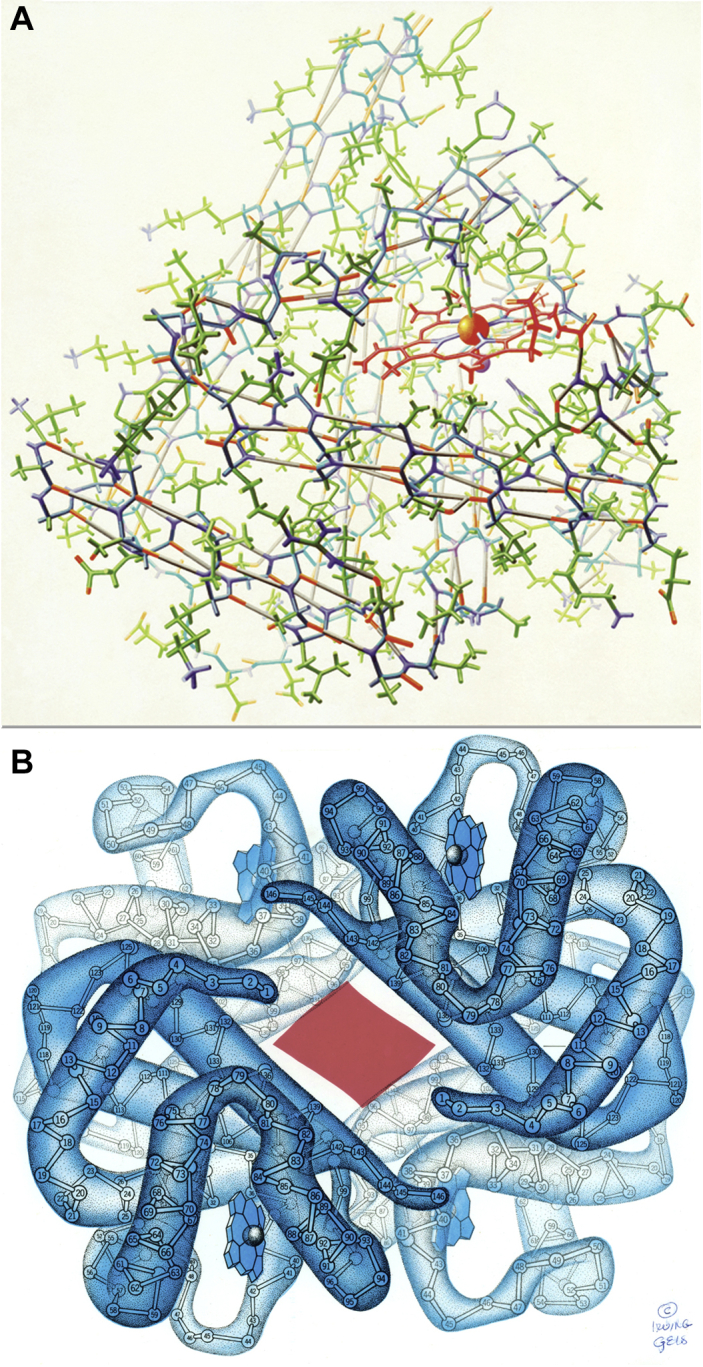
**Pre-PDB protein visualizations by Irving Geis.***A*, painting of the all-atom stick model for myoglobin (including hydrogens), with strong depth cueing and emphasis on the heme ligand and on the helical H-bonds. *B*, lower-resolution representation of the four chains of deoxy hemoglobin, with the numbered Cα-trace inside the smoothed electron density map in *blue*. The *red patch* shows the narrower central opening in the oxy form. Used with permission from the Geis archive at Howard Hughes Medical Institute (www.hhmi.org); all rights reserved.

In the beginning, even before the PDB, visualizations of proteins in scientific reports were mostly either smoothed blobs of density to show helices and connectivity or full-detail Watson–Kendrew brass stick models (1, 2), photographed from the physically built models, and limited to black and white. The professional artist and scientific illustrator who first turned that information into appealing, convincingly understandable drawings was Irving Geis. Figure 1*A* shows Geis' painting of the myoglobin model at 2-Å resolution done for *Scientific American* (3), which conveys the depth cueing and 3D relationships, atom types, H-bonds, and even hydrogens much more effectively than a photograph of the actual brass model. Figure 1*B* shows his representation of the deoxyhemoglobin tetramer (4), with the numbered Cα trace inside a partly transparent, smoothed tube of the low-resolution electron density map. He also drew oxyhemoglobin separately, but since animation was not possible on the printed page, he could only suggest the large conformational change by outlining in red the smaller central opening in the oxy form.

Those myoglobin and hemoglobin structures were what motivated Dave Richardson to take on solving the structure of Staphylococcal nuclease as his PhD project in inorganic (!) chemistry with Al Cotton at MIT, where Jane then joined the group as a technician. Stories about our punch-card days in the 1960s can be found in (5, 6).

Later, when Chris Anfinsen persuaded Jane to take on the task of drawing and classifying all the extant protein structures for Advances in Protein Chemistry (7), she got a number of useful tips from Geis, such as showing backbone and side chain in different colors, and that sometimes inconsistent representations can work better: *e.g.*, Geis' full-bond red for backbone carbonyls and all of the heme *versus* half-bond colors elsewhere (see Fig. 1*A*) and Jane's thick β ribbons *versus* thin α ribbons *versus* round-rope loops (see ribbon figures below). In the other direction, Geis later made a painting for his friend Fred Richards that included a moonlit ribonuclease S in Jane's ribbon style (8). His ribonuclease by itself can be seen in the digital Geis archive at the RCSB (http://pdb101.rcsb.org/sci-art/geis-archive/gallery/rcsb-0002-ribonuclease-s), and the entire painting can be seen in (8), by Richards' grandson Ben Lillie who owns the painting.

**Figure 2:**
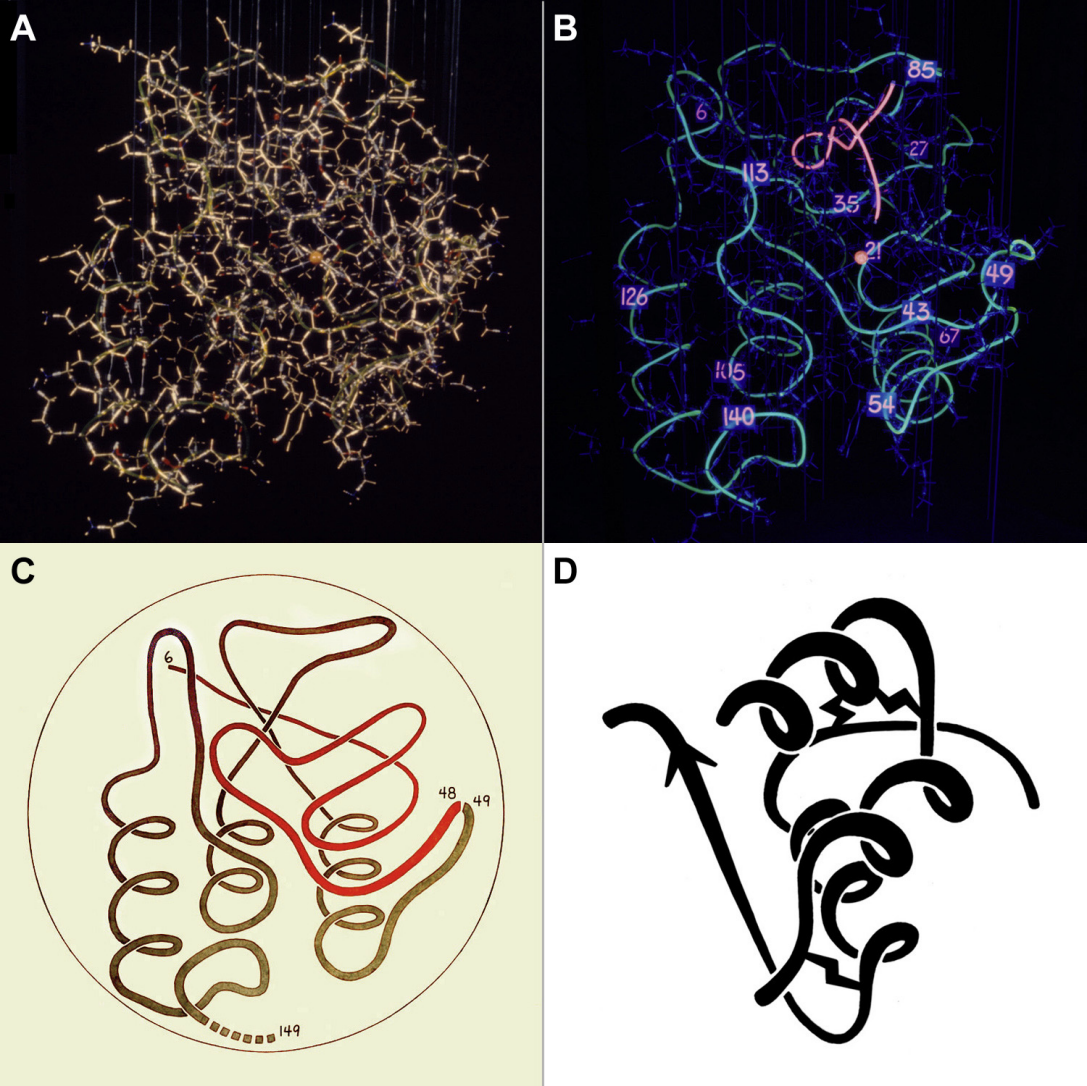
**Preribbon Richardson representations.***A*, photo of the brass model of Staphyloccocal nuclease in room lighting. *B*, photo in UV lighting of fluorescent-dye-filled tygon tubing tied along the backbone of that model. *C*, hand-drawn “worm” of the nuclease backbone, as cleaved into two parts that can still fold up. *D*, more sophisticated worm drawing of the two-chain monomer of insulin.

When Dave and Jane Richardson built a brass model of their Staphylococcal nuclease structure (1sns) (9), they were struggling to show the backbone “fold” clearly. Chris Anfinsen, for whom the nuclease was a disulfide-free model system to study protein folding, suggested we tie 1/4" tygon tubing along the backbone, fill it with fluorescent dye, and view it under UV light. (Fig. 2, *A* and *B*). The result looks much like the earliest computer graphics. Jane made a primitive “worm” drawing from that UV-lit image (Fig. 2*C*), later done in a form more transitional toward ribbons for a few small structures such as insulin (Fig. 2*D*), and Dave learned to draw a worm of the hemoglobin tetramer on the chalkboard for his class. Those were precursors to the ribbon drawings, further inspired by analogy with MC Escher's “ribbon” or “rind” drawings of human heads that alternate a spiral strip of surface with an equal opening, cleverly placed to include just enough of eye, nose, mouth, hair, and shoulders so that you perceive the face as intact and also see the back side of the head (see explanation in (10), or just google “MC Escher ribbon head”). Jane worked until she could see, and then hopefully make viewers see, her β ribbons as continuously H-bonded β sheets, not as a handful of separate arrows.

Not being even an amateur artist, Jane spent an entire year developing effective conventions for the ribbons, practicing how to draw them, and drawing the 75 distinct protein domain structures then known, plus another year writing the long review article they illustrate (7). Fortunately, she was then a nearly invisible Associate whose time was not closely scrutinized except by Dave. She had built or helped build the initial models for four proteins (1sns staphylococcal nuclease, 1gch γ-chymotrypsin, 1sod/2sod Cu,Zn superoxide dismutase, and 1ebx erabutoxin B), and so she was able to visualize in her head the peptide orientations and H-bonding from stereo images of the Cα-trace. The pioneering computer graphics system in Richard Feldman's laboratory at National Institutes of Health in Bethesda allowed interactive choice of viewing direction for a Cα-trace, mono printout at a consistent scale, plus printout of small Cα stereo pairs. Jane drove up there often from Duke, displaying coordinates of different structures either from the PDB or from Feldman's microfiche atlas (11) and bringing home the printouts.

**Figure 3:**
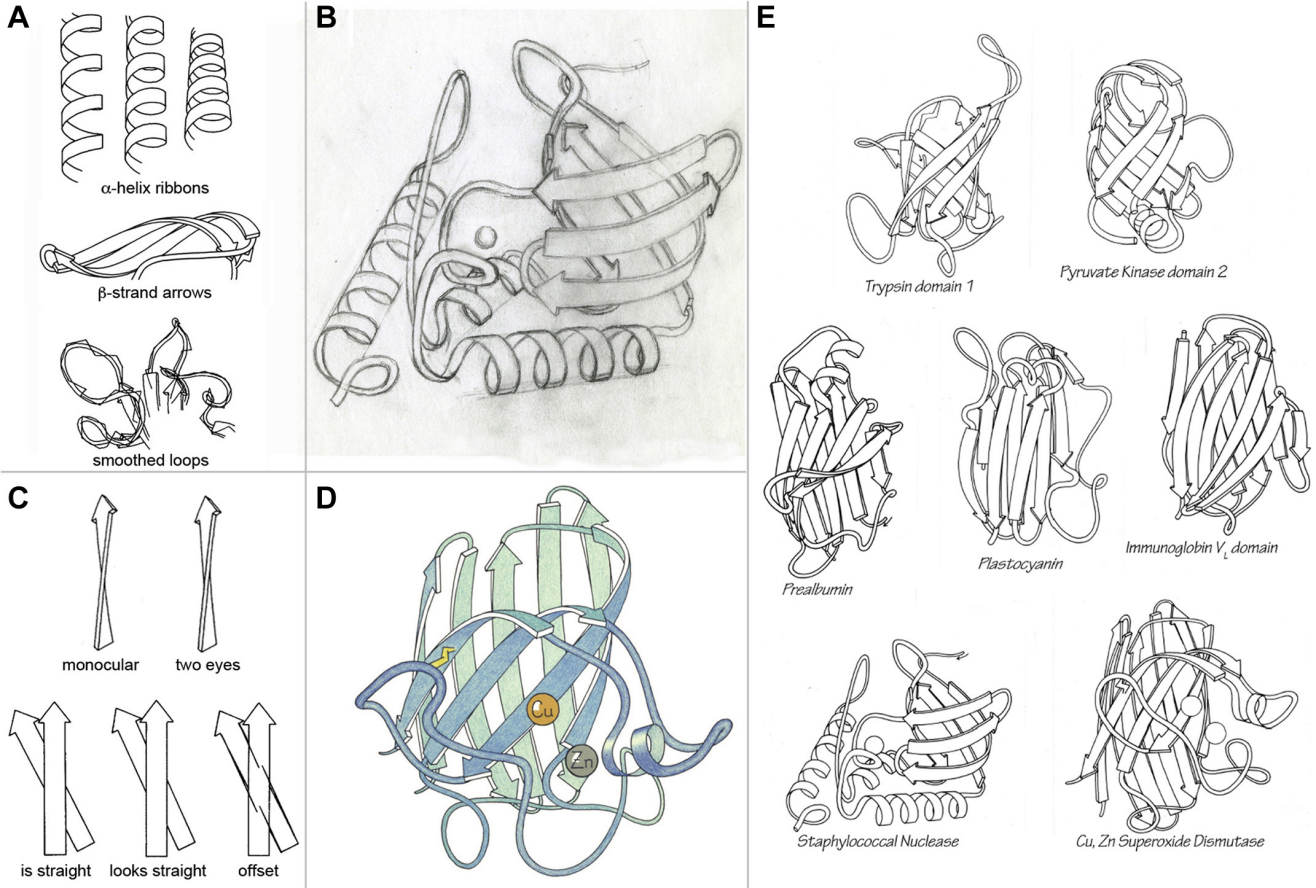
**Jane Richardson's ribbon drawing process as of 1980.***A*, the different conventions for drawing α helix, β sheet, and loops. *B*, the pencil sketch for Staphyloccocal nuclease, done on tracing paper over the scaled Cα plot. *C*, two optical illusions compensated for to draw convincing ribbons. *D*, the pastel-colored ribbon for Cu,Zn superoxide dismutase (SOD) that corrects for both illusions in (*C*). *E*, page 270 from reference (7), including the Greek key folds of Staph. nuclease, of SOD, and of an immunoglobulin V_L_ domain.

The ribbon-drawing conventions for α helix, β sheet, and nonrepetitive loops are shown in Figure 3*A*, with the Cα-trace behind for the loop case; Figure 3*B* shows the pencil sketch for Staphylococcal nuclease on tracing paper over the mono Cα printout; each such sketch involved much trial-and-error erasing and redrawing until it looked right. The final pen-and-ink drawing was done on heavy tracing paper over the pencil drawing, then photographed by Dave to make the high-contrast, touched-up negative used for production of the review article (Fig. 3*D* shows a page of similar Greek-key fold structures) and distributed by us as a coloring book. Looking right also involves allowing for perceptual illusions caused by binocular vision, such as seeing both sides of an edge-on ribbon at once and seeing a bit under each side of the front strand when a strand behind crosses at a low angle (Fig. 3*C*); both of those cases occur in the drawing of our Cu,Zn superoxide dismutase (“SOD”; (12)), shown in Figure 3*D*.

**Figure 4:**
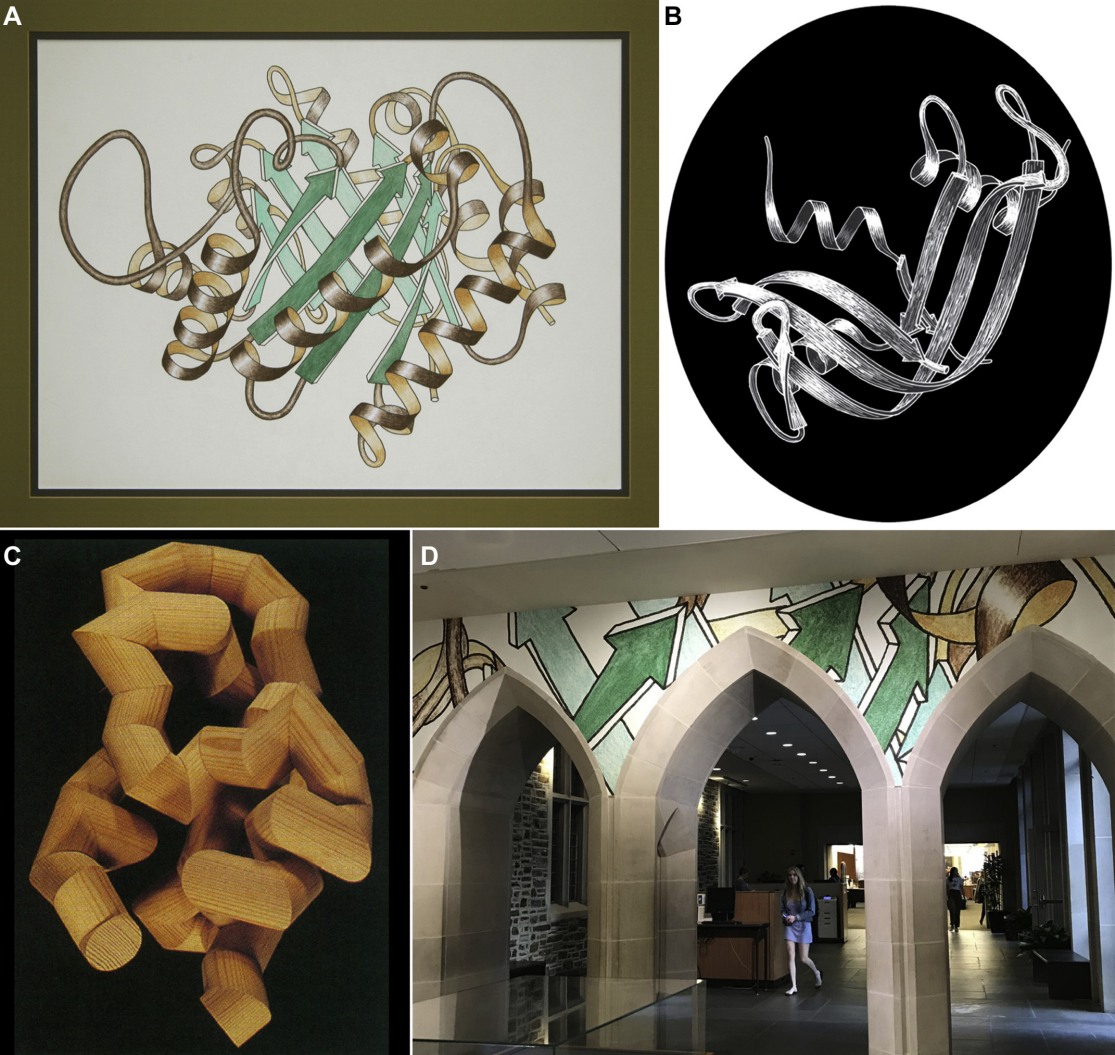
**A gallery of ribbons drawings in other media.***A*, the large, pastel-colored drawing of the (α/β)_8_ “TIM” barrel of triose phosphate isomerase (1tim). *B*, lighted drawing of ribonuclease S, done on scratchboard. *C*, wooden model of expanded Cα-trace for basic pancreatic trypsin inhibitor (BPTI); pieces cut and glued from closet pole. *D*, an enlarged piece from the TIM drawing, on the wall of a Duke Library exhibit about the Richardson's work over 50 years at Duke.

The first shaded versions were done with stick-on plastic cut to fit with an exacto knife, in halftone dots for black and white and in color for slides. Stick figures were often added for ligands or for critical sidechains. Dave's mother, a professional artist (see (13) for an example of her work), encouraged Jane to try other media: pastels, which are wonderful for enlarged versions but were done only for TIM (Fig. 4*A*; 1tim; (14)) and for SOD (Fig. 3*D*) because it is so hard to keep from smudging them; scratchboard (Fig. 4*B*); stained glass; and even a small sculpture of glued-together pieces cut from a 1 ¼-in diameter wooden closet pole with our miter-bevel saw (Fig. 4*C*), which made the cover of *Biophysical Journal* (15). Other people also make ribbon sculptures, such as Byron Rubin (http://5reed.edu/reed-magazine/articles/2019/byron-rubin-protein-sculptor.html), Julian Voss-Andrea (http://julianvossandrea.com), and Bathsheba Grossman (https://bathsheba.com/crystal/). A recent Duke Library exhibit (https://exhibits.library.duke.edu/exhibits/show/invisible/case04) about our work showed that ribbon drawings go surprisingly well with gothic arches (Fig. 4*D*).

Many of our hand-drawn ribbons and our computer graphics images are available with open license on Wikimedia Commons at https://commons.wikimedia.org/wiki/User:Dcrjsr. The TIM barrel drawing (Fig. 4*A*) was Wikipedia Picture of the Day on November 19, 2009. More information and links are available on Wikipedia pages for Ribbon diagram, Kinemage, and Jane S. Richardson.

### Computer graphics: Ribbons and all-atom contact dots

**Figure 5:**
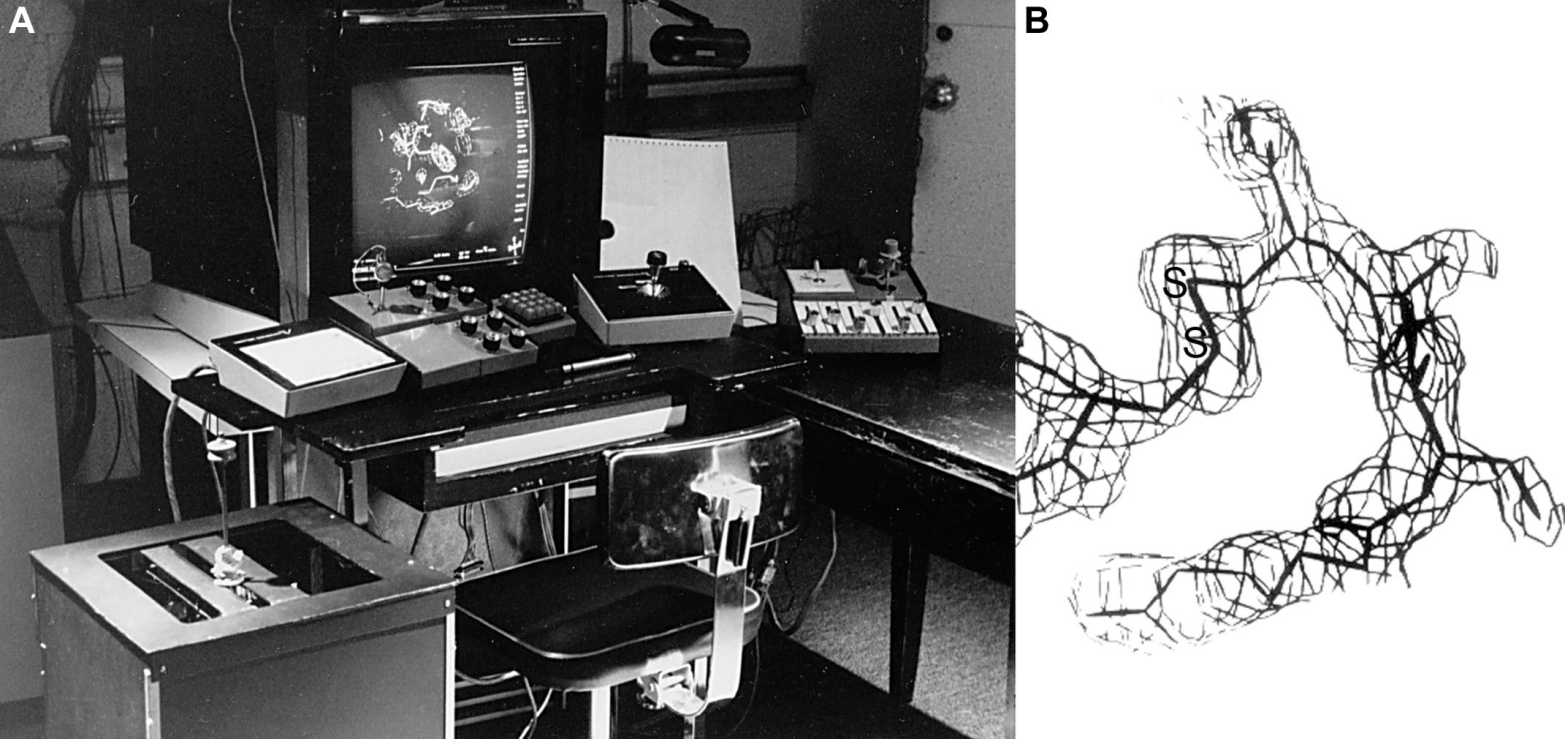
**The GRIP-75 interactive protein model-building system at the UNC computer graphics laboratory.***A*, the user console during a session of fitting a model into electron density, as first done for Cu,Zn SOD. *B*, a closeup of GRIP-75 model and density for the single SS bond in SOD.

Our first exposure to interactive use of computer graphics for crystallography itself was the graphics laboratory in Fred Brooks' computer science department at UNC Chapel Hill. The user console of GRIP-75 (Fig. 5*A*) had both stereo and smooth rotation, and a wonderful array of five knob, slider, and “toothpick” controls, the first computer system capable of fitting an atomic model into an electron density map (16). The calculations were done on an IBM360 that filled the room behind. We were guinea pigs in its development, and our 2-Å resolution structure of Cu,Zn superoxide dismutase (2sod; “SOD”) was the first protein crystallographic model built on computer graphics before building a physical model (17, 18). Figure 5*B* is a snapshot from the GRIP-75 screen, showing the single SS bond in SOD. A parade of users followed until GRIP-75 was gradually superseded by Frodo (19), which could run on hardware accessible to individual laboratories. We stayed on for 20 years, providing driving problems for the UNC graphics laboratory and collaborative graphics projects. That was enormous fun and taught us a great deal about making our own graphics systems.

**Figure 6:**
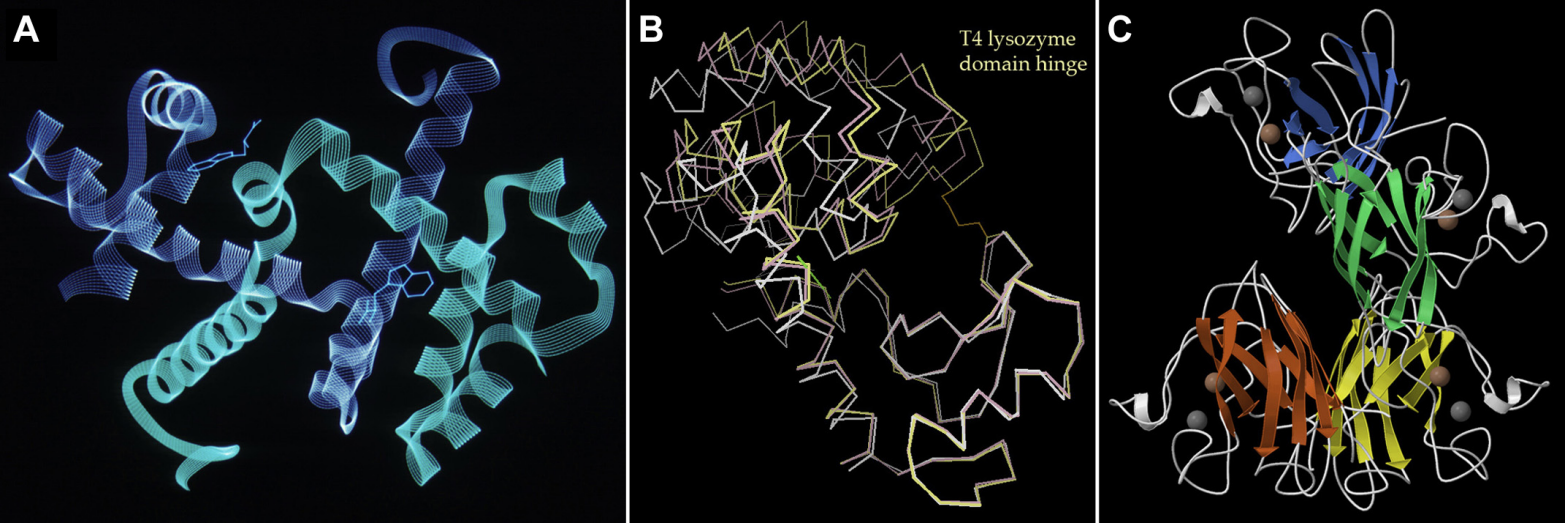
**Dave Richardson's early computer graphics.***A*, multistrand ribbons for the intertwined Trp repressor dimer, on the Evans & Sutherland PS300 calligraphic display. *B*, an animation of conformational change in T4 lysozyme, in his Mage program, for the *Protein Science* journal and the first Apple computers in 1992. *C*, a later Mage display of ribbons for the two SOD dimers in the crystal asymmetric unit (metals as zinc-colored and copper-colored balls).

When we got our own Evans&Sutherland PS300 calligraphic display, people were starting to try out a variety of ribbon-like representations, but most were too literal and choppy. The first version we really liked was in Mike Carson's Ribbons program (20). He gave us his algorithm for smooth B-splines with offsets for helix and turn radius, and Dave coded it as multistrand vector ribbons such as the intertwined dimer of Trp repressor in Figure 6*A*. Then in 1990 we realized that the first Apple home desktop computer could smoothly rotate 500 vectors—enough to show interesting molecular visualizations in 3D or simple animations of conformational change, accessible to anyone. On a hiking trip in the Sierras we worked out the framework for “kinemage” format, including that it is human editable and that any display point clicked on will tell you its identity. Dave then wrote the Mage program to display kinemages, with many interactive viewing and even editing options (21); about the same time RasMol was also developed for home computers (22). Kinemages became a major part of the then-revolutionary electronic supplement to the new *Protein Science* journal, distributed each month on 4K diskettes. The first examples were very simple: an active site or overall Cαs, such as animating the hinge motions of T4 lysozyme domains to illustrate a paper in that journal issue (Fig. 6*B*). As the capabilities of personal computers rapidly evolved, larger models and even ribbons could be shown interactively (Fig. 6*C*).

**Figure 7:**
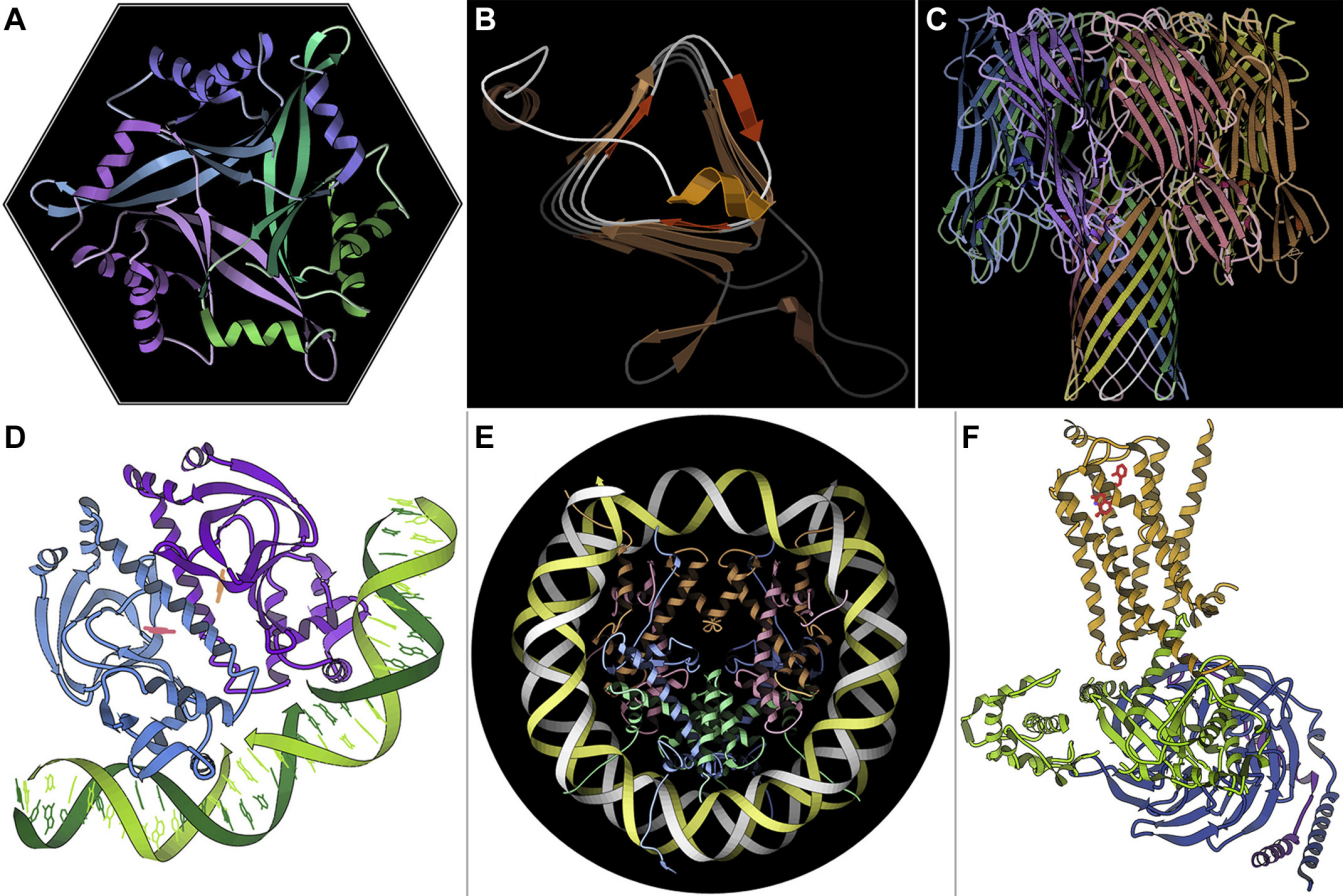
**A gallery of computer-drawn ribbons, the first two from Mage and the rest from KiNG.***A*, interdigitated trimer of human CUTA, with each chain in a different color; 1xk8. *B*, three-sided, *left*-handed β helix of an archaeal carbonic anhydrase, emphasizing the helical loop that protects this end from β–β aggregation; 1qre. *C*, α-Hemolysin 7-mer, with its transmembrane β-barrel pore at *bottom*; 7ahL. *D*, CAP protein dimer binding and bending DNA; 1cgp. *E*, nucleosome, with central core of histone proteins (*green*, *blue*, *peach*, *pink*) encircled by two turns of DNA (*yellow*, *white*); 1aoi. *F*, seven-helix transmembrane β2-adrenergic receptor (*gold*) with its in-cell signaling G protein chains (*green*, *blue*, *purple*); 3sn6.

These days, ribbon graphics are limited in size and complexity mainly by what can be usefully perceived by the viewer, rather than by what can be calculated and displayed interactively, and other new representations have taken over for very large molecules, complexes, and even whole cells (see the Goodsell perspective). A gallery of some favorite mid-size ribbons of proteins and complexes is shown in Figure 7, *A*–*F*, from a small trimer to protein–DNA complexes and membrane proteins. The first two of these were created in Mage, and the rest in KiNG (23, 24), a Java kinemage viewer for online as well as local use and with many additional editing and remodeling features. An interesting sideline is that the kinemage format is not limited to molecules but is expressed as geometric primitives (line, point, triangle, etc.). Kinemages have been made for social networks, food webs, geodesic domes, a map of the world, etc. One example plotted measurements of a vulture flight path in 3D, which unexpectedly showed that vultures do not actually circle but fly in squared-off patterns parallel and perpendicular to wind direction, just as a small plane would.

### All-atom contact analysis

After working on early protein design, which could then achieve correct secondary structures and topologies but not well-ordered native-like structure (15), we realized there was one of the inherent problems we could work to overcome: that if we changed from the standard of implicit “united-atom” treatment to including all explicit H atoms, and then paid attention to their contacts, we could provide a realistic, quantifiable, and visualizable analysis of detailed atom–atom internal packing. The atom–atom contact dots we use are more or less the inverse of Connolly dots that outline the accessible surface of molecules (25). Our Reduce program places all explicit H atoms and optimizes their rotations and interaction networks for H-bonds, van der Waals (vdW), and clashing overlaps (26). This all-atom contact analysis visualizes how the atomic surfaces interact, rather than traditional energetics that is pairwise from atom centers and is only numerical, not visual.

**Figure 8:**
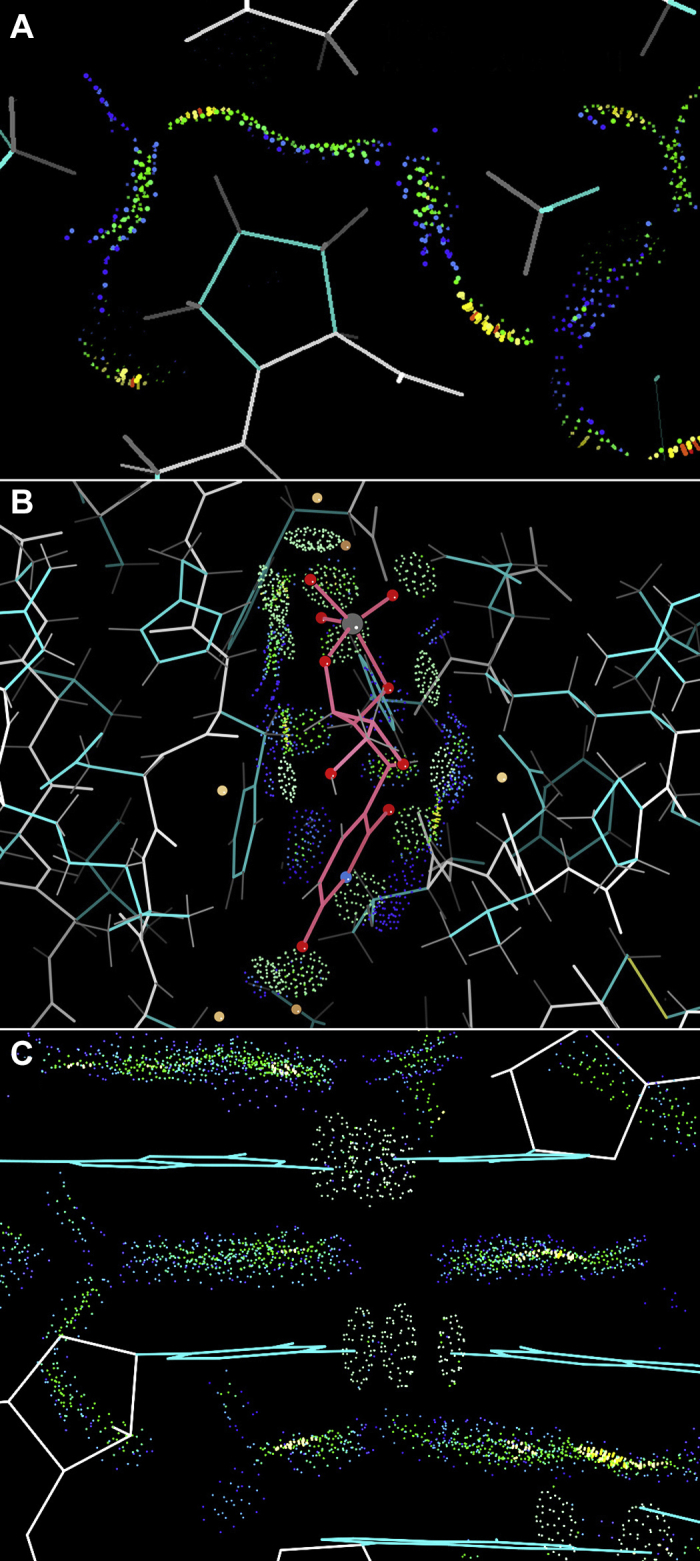
**Using all-atom contact dots to understand an interaction.***A*, well-matched, interdigitated, hydrophobic van der Waals surfaces of the explicit hydrogens on a proline ring and its neighbors in elastase at 1.5 Å; 1ezm. *B*, uridine vanadate transition-state inhibitor (*pink*) in ribonuclease A at 1.3 Å is tightly held by 13 good H-bonds; 1ruv. *C*, large areas of van der Waals contact for stacked base pairs in a G-riboswitch at 1.35 Å; 4fen.

Figure 8*A* shows the undulating, interdigitated dots for vdW contacts around the H atoms on a proline sidechain at high resolution (1ezm; (27)). Green dots are for perfect atom–atom contact, blue for very close but not quite touching, and yellow for just a bit tight. Figure 8*B* shows a transition-state inhibitor held very tightly in ribonuclease (1ruv; (28)) by 13 good H-bonds (pillows of pale green dots). Figure 8*C* shows the base-stacking vdW contacts and base-pair H-bonds in an RNA double helix of a mutant G riboswitch at 1.35-Å resolution (4fen; (29)). The all-atom contact system is confirmed by the fact that the well-ordered parts of high-resolution experimental structures have almost no clashes, good density of H-bonds, and interdigitated hydrogens to give excellent vdW contacts. That system also provided a new and powerful way to find misfit local conformations in the experimental models, primarily by “clashes,” defined as unfavorable overlaps ≥0.4 Å. That discovery motivated our establishment of the MolProbity validation web service, discussed below.

### RNA in seven dimensions

**Figure 9:**
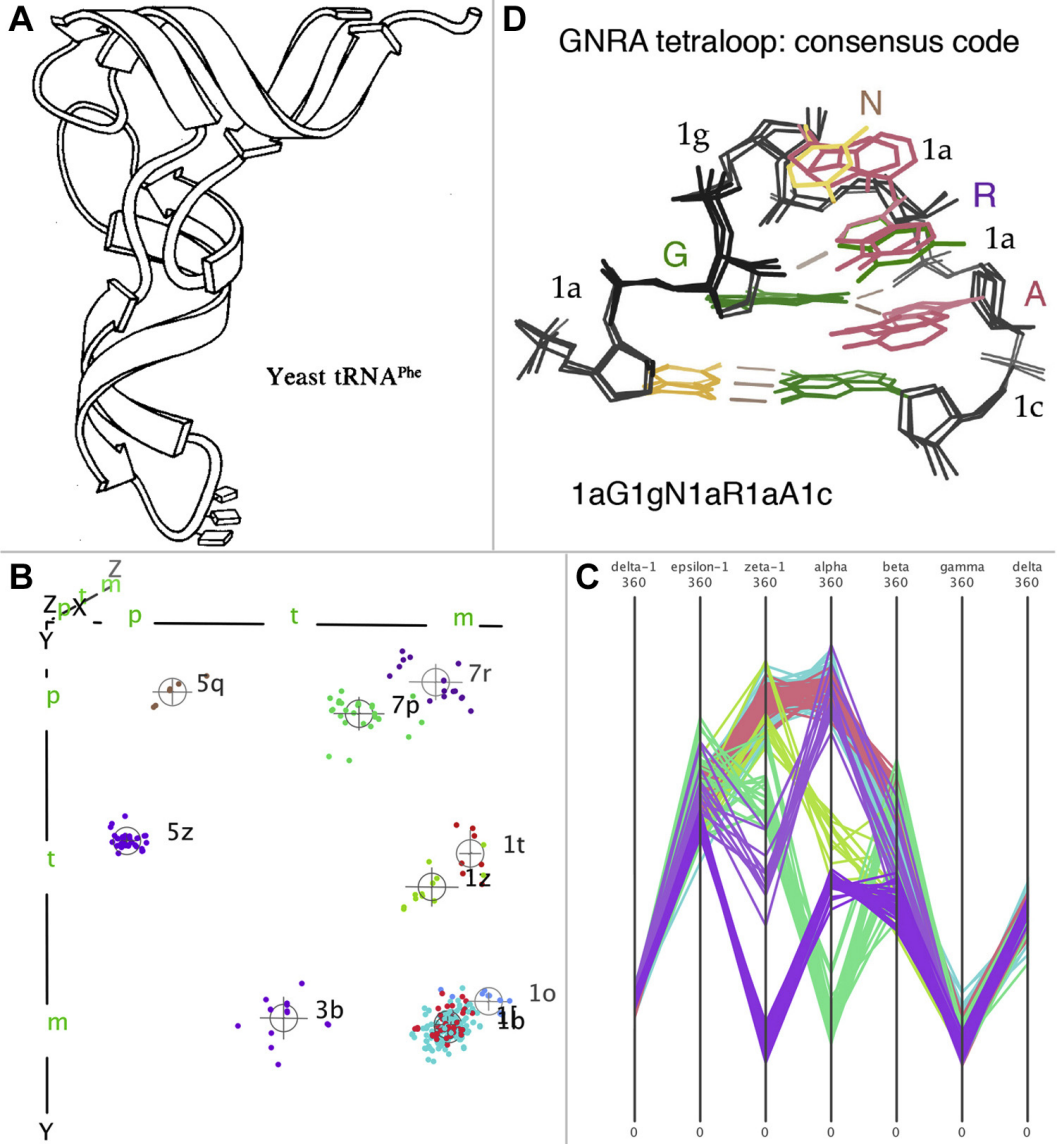
**Visualizations of RNA structure and backbone conformations in 3D and 7D.***A*, hand-drawn ribbon of yeast tRNA Phe, with its two pairs of stacked stem-loops and the anticodon triplet bases at the *bottom*. *B*, ten colored and labeled clusters in the ζ, α, γ choice of 3D view for seven-dihedral RNA backbone “suite” conformers (the **1b** and **1[** clusters and their labels overlap completely in this view, because they differ only in the β dimension); displayed in KiNG. *C*, the same ten clusters shown in parallel coordinates, accessible by clicking the “/” key. *D*, three superimposed examples of GNRA tetraloops labeled with their two-character conformer names alternated with their base identity, thus neatly describable as 1aG1gN1aR1aA1c.

After the first ribosomes came out in 2000, our new student Laura Murray led us into revisiting our early fascination with RNA at MIT in the 1960s, when our friend Sung-Huo Kim solved the first structure of a complexly folded RNA (Fig. 9*A*), dramatically revising expectations about RNA structure beyond flat secondary-structure layouts such as the tRNA “cloverleaf.” The ribosome added more than an order of magnitude more data, enabling a realistic analysis of the hard-to-see and inherently high-dimensional RNA backbone conformations. There are six variable dihedral angles in a PO_4_-to-PO_4_ RNA nucleotide, but we discovered that the seven-dihedral sugar-to-sugar suite parsing of the backbone shows better clustering because it includes more backbone interaction than the nucleotide as well as being directly influenced by the interaction of successive bases. After trying a system of cross-picking between two 3D windows, Dave in Mage and Vincent Chen in KiNG developed an integrated high-dimensional technique for displaying and analyzing that data. It works by defining “views” for specific triples of parameters, choosing and coloring the points in an apparent cluster (Fig. 9*B*), and then checking cluster validity in other 3D combinations and also in parallel coordinates (Fig. 9*C*). That work culminated in a collaboration of five different laboratories to agree on a consensus library of RNA backbone conformers with two-character names or **!!** (bang-bang) for outliers (30). The 7D graphics are for doing the research, whereas the results allow anyone to meaningfully name and visualize an RNA motif such as a stack switch, an S-motif, or a GNRA tetraloop (Fig. 9*D*). When combined with all-atom contacts, such an image is even more informative and can guide correction of RNA validation outliers.

### Virtual reality in the DiVE: NMR RDC curves

**Figure 10:**
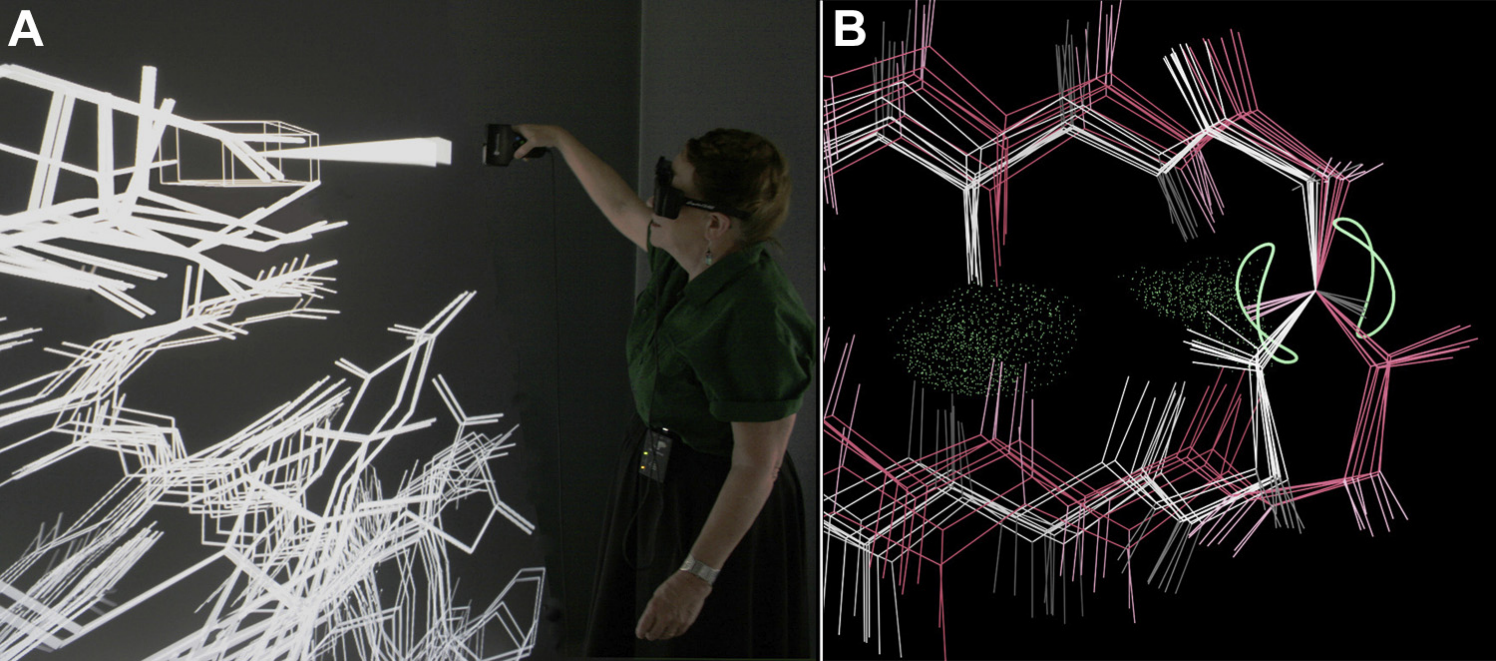
**Doing macromolecular research in immersive VR (virtual reality).***A*, Jane selecting one bond of a multimodel NMR ensemble, using an early version of our software in the Duke DiVE. *B*, in KinImmerse (31), the NMR ensemble of 2jng cocentered on a backbone N atom to analyze fit to the elegant two-branch curve (*pale green*) that represents an experimental RDC (residual dipolar coupling) measurement. In this case, the NH atoms of the *pink* models lie on the wrong branch of the curve and the *white* ones are correct (see text).

The DiVE (Duke immersive Virtual Environment) 3-m^3^ virtual reality lets us explore whether immersive virtual reality could be set up to enable molecular research as well as to enhance intuition through a compelling visual and interactive experience. Working with the Visualization Technology group at Duke, we jointly programmed KinImmerse display of the kinemage format in the DiVE, with atom selection, identification, and measurements (Fig. 10*A*; (31)). Our test case was bringing to NMR structural ensembles the direct visual comparison of model to data that we rely on in crystallography by looking at the model within the electron density map. Basic NMR data measures very local atomic relationships: atom-pair distance by nuclear Overhauser effect (NOE) values and atom-pair orientation to the magnetic field by RDC values, and so is well suited to an aligned and zoomed-in perspective within the ensemble. We provided that perspective by a simple but novel tool called cocentering (on a specific atom type, which can be toured through the sequence) to then compare in each residue how well each model fits the relevant local data. NOE data are easily represented as a vector between the two specific atoms, but an RDC (residual dipolar coupling) measurement means the atom–atom orientation lies somewhere along a pair of curves. Those curves can be drawn around one of the atoms in the ensemble of models (such as the backbone N for an NH RDC), but that was never done until cocentering made it understandable. Figure 10*B* shows the elegant RDC curves around the cocentered N of Glu 36 in 2jng. In this case some of the models must be wrong, since the H atoms should all be on the same branch of the RDC curve. Which group of models is correct (the white ones) can be decided by other validation criteria such as H-bonding, clashes, and φ,ψ values. This system was later ported back to single-screen viewing, but might not have been thought of there.

### Validation markup and corrections, including SARS-CoV-2 molecules

**Figure 11:**
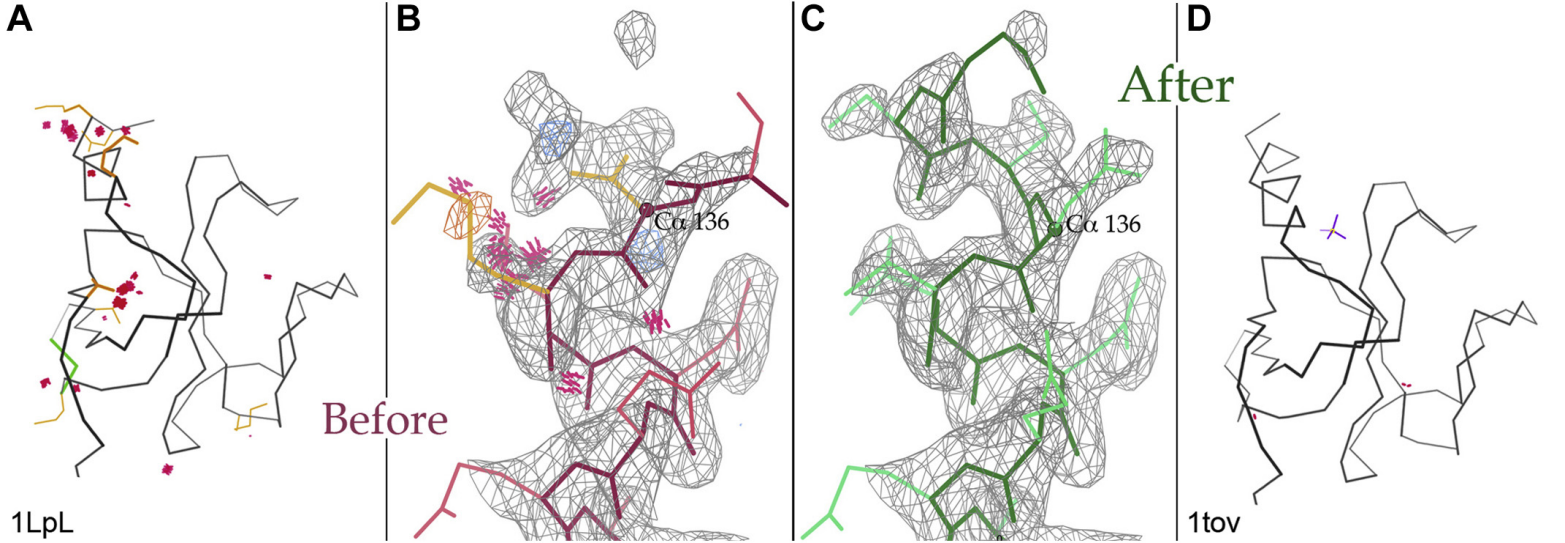
**Using MolProbity validation outliers to guide structure improvement.***A*, Kinemage display of all-atom clashes (clusters of *hot pink* spikes) and sidechain rotamer outliers (*gold*) in context of the Cα backbone in 3D, for 1LpL. *B*, zooming in on the largest group of outliers, along with full model, electron-density map (*gray*), and difference density peaks (*blue* and *orange*). At Cα 136, the first residue modeled, density extends well beyond the Asp sidechain and is too short for the backbone. *C*, after correction of that sidechain–mainchain switch, the outliers are gone and another turn of helix can be modeled. *D*, the final result (deposited as 1tov) has only two small clashes, a new sulfate, and a 4% lower Rfree.

Our MolProbity web service adds other validation measures to the all-atom contacts (bond length, bond angle, and Cβ deviations, Ramachandran φ,ψ dihedrals, sidechain rotamers, and RNA sugar puckers and backbone conformers). In addition to sequence-based charts and summary statistics, it outputs visual markup for the local outliers on an interactive image of the 3D model. Each type of outlier has a distinct color and shape, and they stand out clearly on a simple Cα-backbone model (Fig. 11*A*). The user can then zoom in and overlay the electron density map to see the full details, especially for a cluster of multiple outliers close in 3D, such as the helix start in Figure 11*B*.

MolProbity gradually became considered state of the art in model validation. However, model validation, if considered just as flagging problems, would be a very depressing and unpopular activity—what makes it positive and even exciting is the prospect of helping to fix many of those problems. The cluster of clashes and rotamer outliers in 1LpL (32) at upper left in Figure 11*A*, and expanded in Figure 11*B* along with the map and difference-density peaks, was diagnosed as an incorrect switch of backbone *versus* sidechain at the 136 Cα atom. Local rebuilding with the correct switch choice at 136 and alternate conformations for Lys 137 removed those outliers and enabled building an additional turn of α helix into the previously unoccupied density (Fig. 11*C*). Our proof-of-concept correction of this 1.77-Å structure lowered the R_work_ by only 1% but the R_free_ cross-validation measure by 4% (33), indicating that the model changes are true improvements: only two tiny clashes and a new sulfate replacing a tight cluster of waters (Fig. 11*D*).

Over the past 20 years, this system of validations and visualizations has enabled both individual structural biologists and automated programs to significantly improve the quality of worldwide PDB (wwPDB) depositions at the most common resolutions from sub-1 to about 2.5 Å. Recently, however, both X-ray crystallography and most especially cryo-EM (34) have succeeded in solving large numbers of huge, dynamic “molecular machines,” mostly at resolutions poorer than 2.5 Å. These new structures make revolutionary contributions to our knowledge of biology, but that lower resolution introduces new systematic errors insensitive to traditional validation metrics. We and others are developing new types of validation that can detect local errors even when the traditional outliers have been artificially refined away, often pushing them to a legal but incorrect conformation. Our most effective new metric so far is CaBLAM, which tests whether the modeled peptide orientations are consistent with the surrounding five-residue stretch of Cα backbone (35).

**Figure 12:**
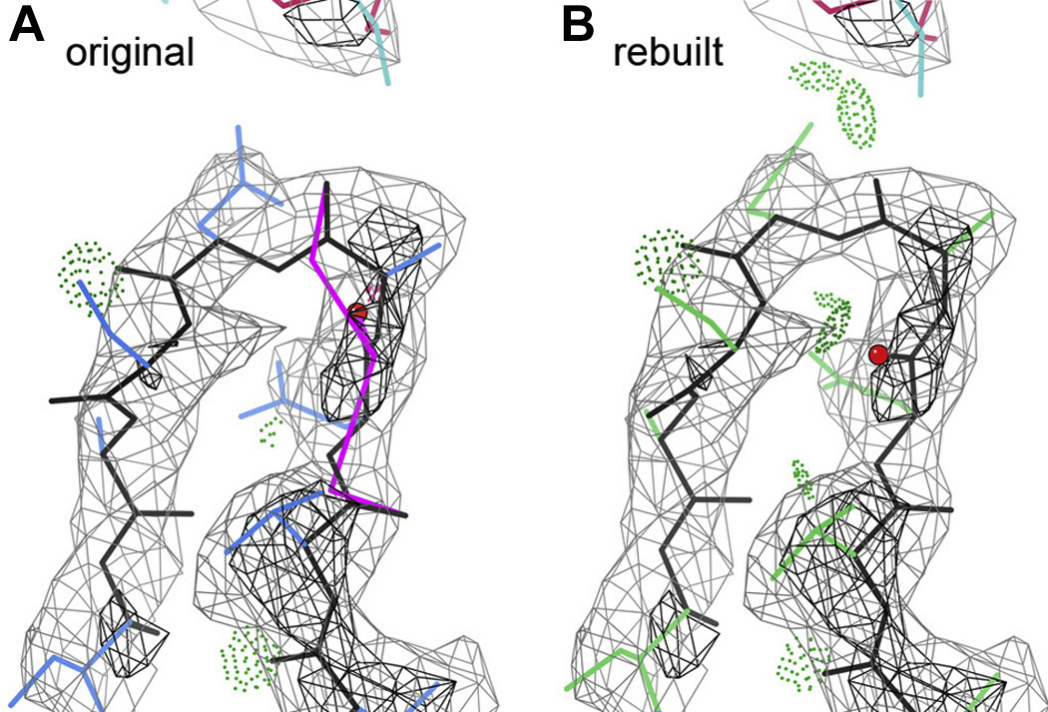
**CaBLAM outliers to flag problems at 2.5 to 4 Å, applied to SARS-CoV-2 structures.***A*, original model of a loop in Nsp8 of the 7bv2 RNA-dependent RNA polymerase structure, with two adjacent CaBLAM outliers and only three H-bonds. *B*, with a large reorientation of the central peptide of those outliers, this loop forms a near-ideal β-hairpin with seven H-bonds including two to an adjacent chain in the complex (7bv2 v2.0).

In 2020, structural biologists mobilized to produce and release structures of the SARS-CoV-2 molecules with unprecedented speed, and we used all of our tools to identify the occasional errors at important sites in those structures that were provably correctable, in collaboration with Tristan Croll and his ISOLDE program described below. The problems included stretches of sequence misalignment, ions with the wrong charge, a mismodeled SS bond that distorted the interface with an antibody, and many individual peptide reorientations or *cis*–*trans* isomerizations that mattered. Visual communication was essential to the success and speed of this process, by convincingly illustrating the recommended changes to the depositors, encouraging them to make those changes in their own models and update their PDB entry. Recently the PDB made it possible for the depositor to update coordinates with a new version number not a new PDB code. They prioritized the new SARS-CoV-2 versioning, and the whole process took just a few weeks from the initial release to an improved model available for download (36). Figure 12 shows an example of documentation for a simple change, where two successive CaBLAM outliers (magenta lines) suggest a large rotation of the central peptide (red ball on the carbonyl O), allowing the loop to settle into a near-ideal β hairpin and enabling two strong H-bonds to another chain in this polymerase complex that produces the new viral genomes (7bv2; (37)).

**Figure 13:**
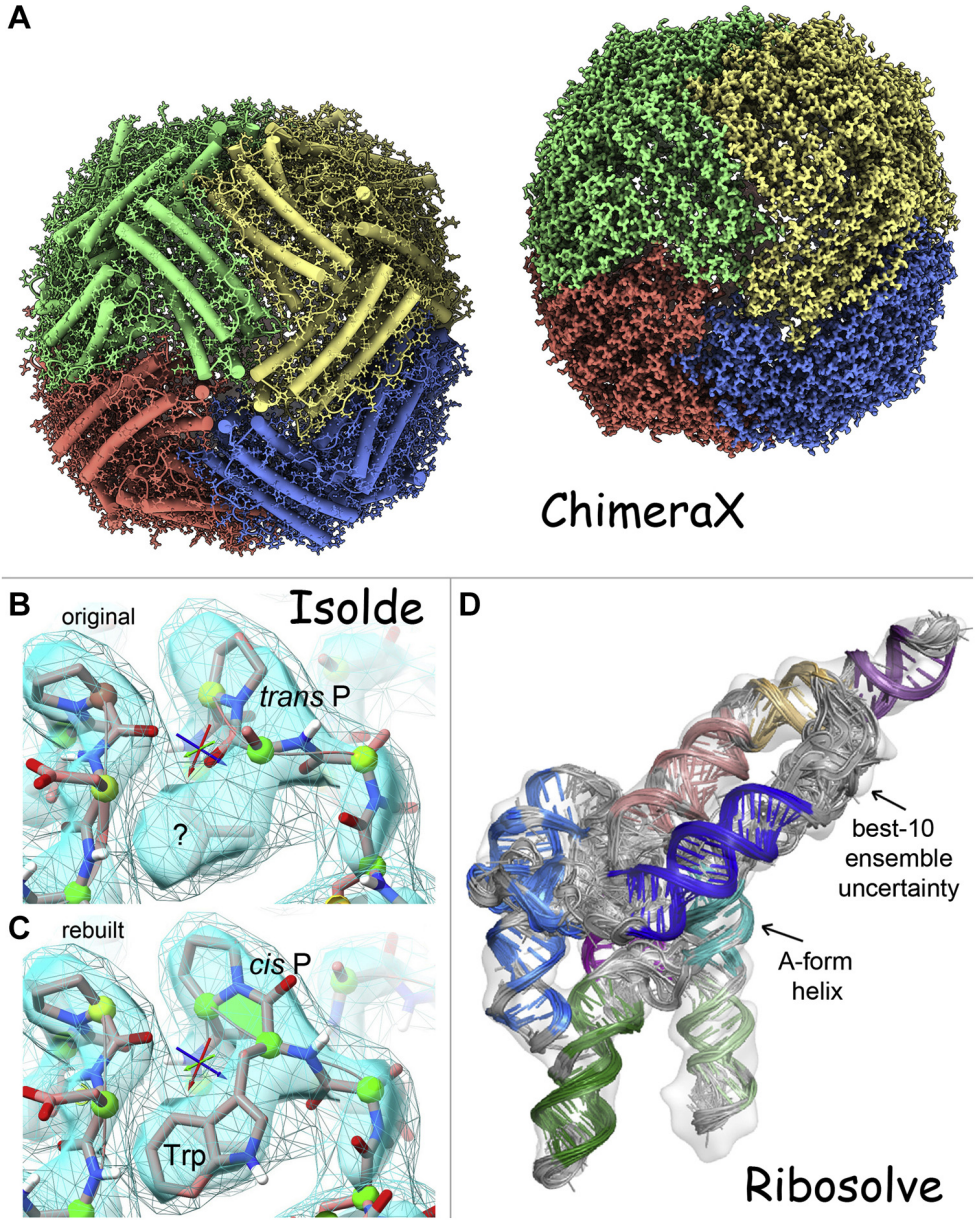
**Favorite current visualization types.***A*, from ChimeraX, fast-ambient-occlusion rendering of surface shape and shadows for helix-cylinders or density map surface of apoferritin in 7a4m at 1.23-Å resolution. *B* and *C*, from ISOLDE user-guided molecular dynamics on the 7bv2 SARS-CoV-2 polymerase, correction of a *trans* Pro in *B* that should have been *cis*, allowing the adjacent Trp sidechain to occupy its very clear density in *C*. *D*, a visualization of model uncertainty from the new Ribosolve pipeline that distinguishes fairly clear A-form helix from uncertainty (*gray*) in the best ten diverse Rosetta models out of thousands for the hc16 ligase product cryo-EM structure (6wLn), at 10-Å resolution and with an estimated ensemble accuracy of 6.3 Å.

### Favorite current visualizations

• Fast-ambient-occlusion in ChimeraX (38): Their recently implemented fast-ambient-occlusion computer rendering provides beautifully and comprehensibly shaded surfaces that can be perused at leisure or smoothly rotated in real time for atomic models as big as a ribosome, or for the complex detail of the density-map surface of 7a4m apoferritin at 1.23-Å resolution (39) seen in Figure 13*A*.
• Tristan Croll's visual model rebuilding in ISOLDE (40), which thoroughly achieves what we were trying to do decades ago (41): Its user-guided molecular dynamics in context of the density map and with continuously updated validation flags (Fig. 13, *B* and *C*) provides a uniquely powerful way to improve protein model quality at resolutions poorer than about 2.5 Å, including problems ranging from individual *cis*-*versus*-*trans* peptides to regions of sequence misalignment (Croll 2021).
• Rhiju Das' visualization of RNA model uncertainty (42): The Ribosolve pipeline output includes a visual ensemble of the top ten sufficiently distinct models out of thousands (by a pretty realistic Rosetta score), represented as shown in Figure 13*D*. Ensembles are common, but their breadth is usually either unrealistically tight or unrealistically broad. The especially challenging aspect going forward will be learning to separate actual motion from model ambiguity and then visualizing those separate components.
• Our all-atom contacts in KiNG: These dot-contact images (see above) are still the best way to show, correct, and comprehend the details of atom–atom contact within or between molecules.
• David Goodsell's representations: For individual proteins and complexes such as the RCSB's “Molecule of the month” series (which we helped make available open-license on Wikimedia Commons and from there in many Wikipedia articles), they convey surface shape especially well. For larger molecular environments, his drawings changed the perceptions of both scientists and laymen to appreciate how crowded a cell really is.

## The Goodsell perspective

When I was applying for graduate school in 1982, I had the great fortune of having abundant material to spark my interest in structural biology. At the time, the PDB archive contained just over 100 structures. A trip to the library yielded inspiring books like Dickerson and Geis “Structure and Action of Proteins” (4). During one of my interviews for graduate school, one of the professors handed me her well-thumbed photocopy of Jane’s “Protein Anatomy,” and after reading that cover to cover, I knew that I wanted to pursue a career in structural biology.

**Figure 14:**
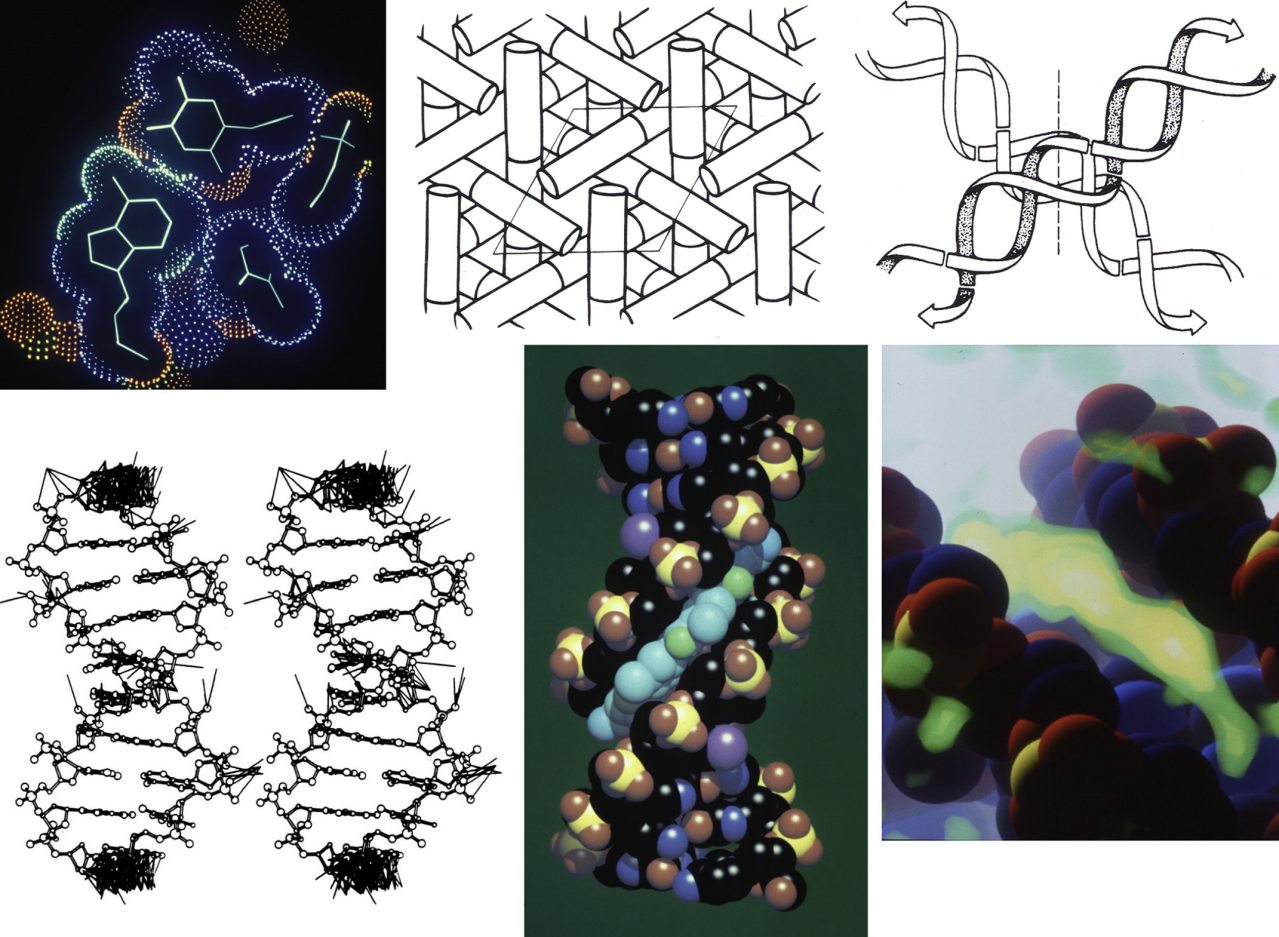
**Early experiments in molecular graphics.***Top*, dot surface and covalent skeleton displayed interactively with the Evans and Sutherland MultiPicture System with custom software. Two hand-drawn illustrations of packing of DNA decanucleotides in crystal lattices. *Bottom*, stereo pair of a bond diagram created using ORTEP and output on a pen plotter, with extra lines depicting the contacts between neighboring molecules in a crystal lattice. Raster image of a drug complex with DNA using shiny Phong shading, and volume-rendered image of electron density of a drug in the minor groove of DNA, both created with custom computer graphics software.

It was a very exciting time to be in structural biology, and in visualization of structural biology in particular. Affordable hardware for computer graphics was just then becoming available, at a cost that made it accessible to individual laboratories or departments (43). For example, during my graduate work with Richard E. Dickerson at UCLA, I had ready access to an Evans and Sutherland MultiPictureSystem, various pen plotters, and down the hall, a (quite slow) AED raster display. Amazingly, apart from occasional ORTEP diagrams on the pen plotter, this technology was virtually unused. This was no surprise, since effective software was still being developed and was largely the domain of visualization experts. So I started looking for available programs, and also writing my own programs, for displaying and presenting the DNA structures that the laboratory was determining (44). Also, artwork with traditional media has always been a big part of my life, so when the topics were too complex for my budding Fortran skills, I drew diagrams by hand. Two examples are included at upper right in Figure 14.

After meeting Arthur J. Olson at a meeting of the Molecular Graphics Society, I moved to the Scripps Research Institute to focus on computational biology and molecular graphics for my postdoctoral work. Art’s laboratory, to this day, is highly exploratory, using the newest developments in graphics hardware to explore biomolecules in fresh, new ways. When I joined, he had built a strong body of work in molecular animation and in representing biomolecules with surface-based approaches, and in the years since then, he has explored new media such as 3D printing, virtual reality, and augmented reality. During my postdoctoral work, I added some methods for volume rendering and also started work on nonphotorealistic cartoony methods of rendering (45). Perhaps most importantly, I also became an active member of a creative community of structural biologists, computer scientists, and scientific artists all focused on revealing the wonders of the biomolecular world. This has led to my life-long interest in depicting the size and shape of molecules, and from that, how they function in living systems.

### Surface metaphors to explore shape and size

What does a molecule look like? This question has fascinated structural biologists ever since it became clear that molecules have a defined structure, and an active community of artists and scientists have charted an ever-changing path through answering it. The central challenge, of course, is that this question has no direct answer. Given the physics of light and the scale of molecules, we can never “see” an individual molecule, at least as anything more detailed than a point of light. Instead, we are forced to create metaphors for what we might see if the physics were more amenable and use these metaphors to synthesize images of molecules. It is in these metaphors that the fun begins, since the goal is to create an image that somehow captures the properties and personality of the molecule, so that the image will provide a way to learn more about the molecule, its structure, its interactions with other molecules, and ultimately, its function.

As Jane and Dave discuss above, a host of effective metaphors have been used for exploring the internal structure, folding, and detailed atomic interactions of biomolecules, most notably, Jane’s ribbon diagrams and many flavors of covalent bond representations. Most of my work has focused on the overall shape and size of molecules and how they fit together into higher-order assemblies and ultimately into functional viruses and cells. Space-filling metaphors and their many variants are my favorite way to explore these aspects of biomolecules. The power of this metaphor was amply demonstrated by its creator, Linus Pauling, who used it, among other things, to develop the detailed model of the alpha helix and its structural constraints (46). Today, all molecular graphics programs provide options for creating representations of the steric surface of a molecule. A few have shown lasting utility, and a few are seeing increased use as structural biology moves to larger and larger assemblies.

Linus Pauling’s original concept was to center a sphere on each atom, with a size that represents the typical contact distance between noncovalently interacting atoms. When combined with a standard coloring scheme, this simple idea provides a powerful and flexible method for representing the steric bulk of a molecule and understanding how they exclude or fit together with other molecules. The simplicity of the approach also makes them quite easy to incorporate into molecular graphics software.

**Figure 15:**
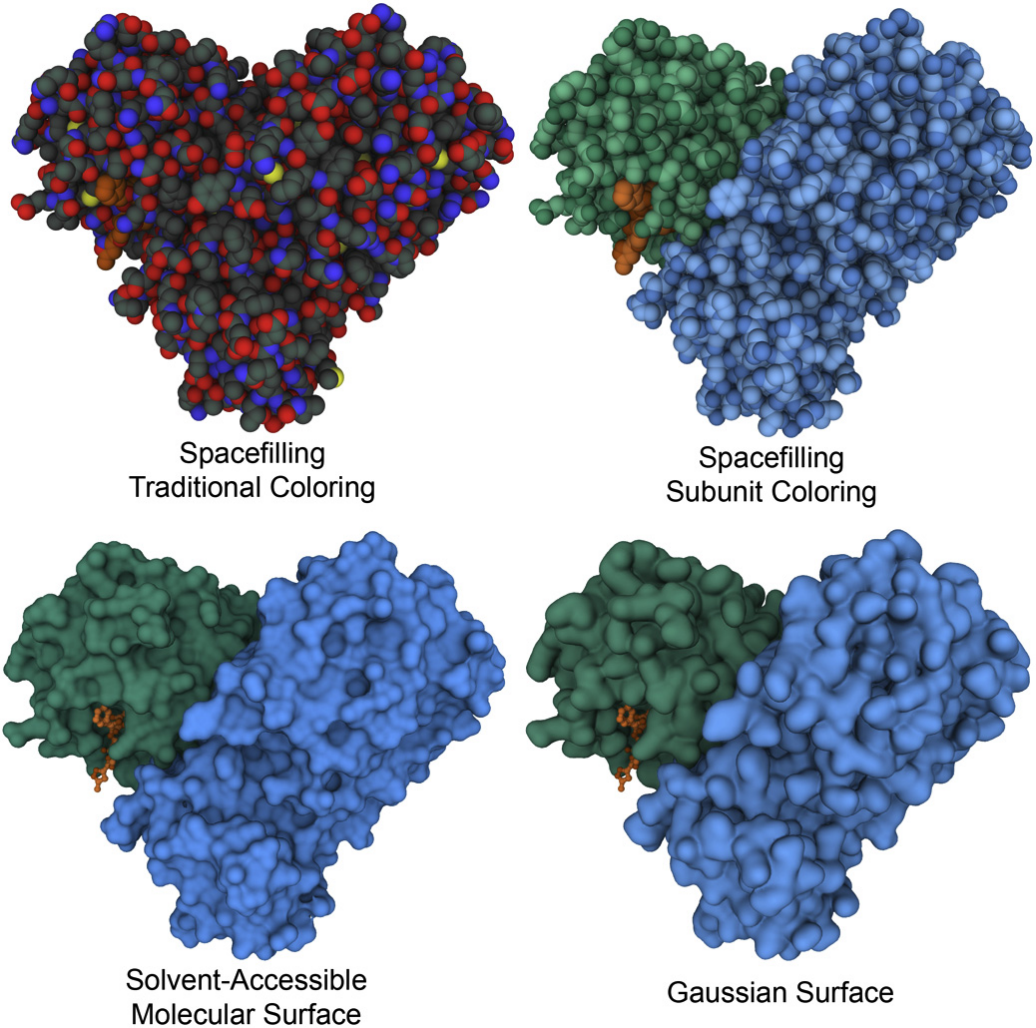
**Several common surface representations used to display biological molecules.** The coronavirus main protease is shown, with an inhibitor in *orange* blocking the active site (6Lu7; (66)). All images created with Mol∗ (67) at the RCSB PDB website (rcsb.org).

A variety of specialized surfaces were proposed in the 1970s and 1980s to explore in more detail the way macromolecules interact (Fig. 15) (25, 47). These surfaces are generated by rolling a probe atom everywhere around the molecule and saving up all the points that it touches. Most often, the probe represents a water molecule, creating a “solvent-accessible” surface. These surfaces have many uses, such as defining the shape of ligand-binding sites and protein–protein interfaces and providing a quantitative measure of water interaction area for use in hydrophobicity calculations. Additional variants are possible for specialized tasks, for example, saving the surface drawn out by the center of the probe sphere or using spheres of multiple sizes to evaluate a “fractal dimension” of the surface (48).

**Figure 16:**
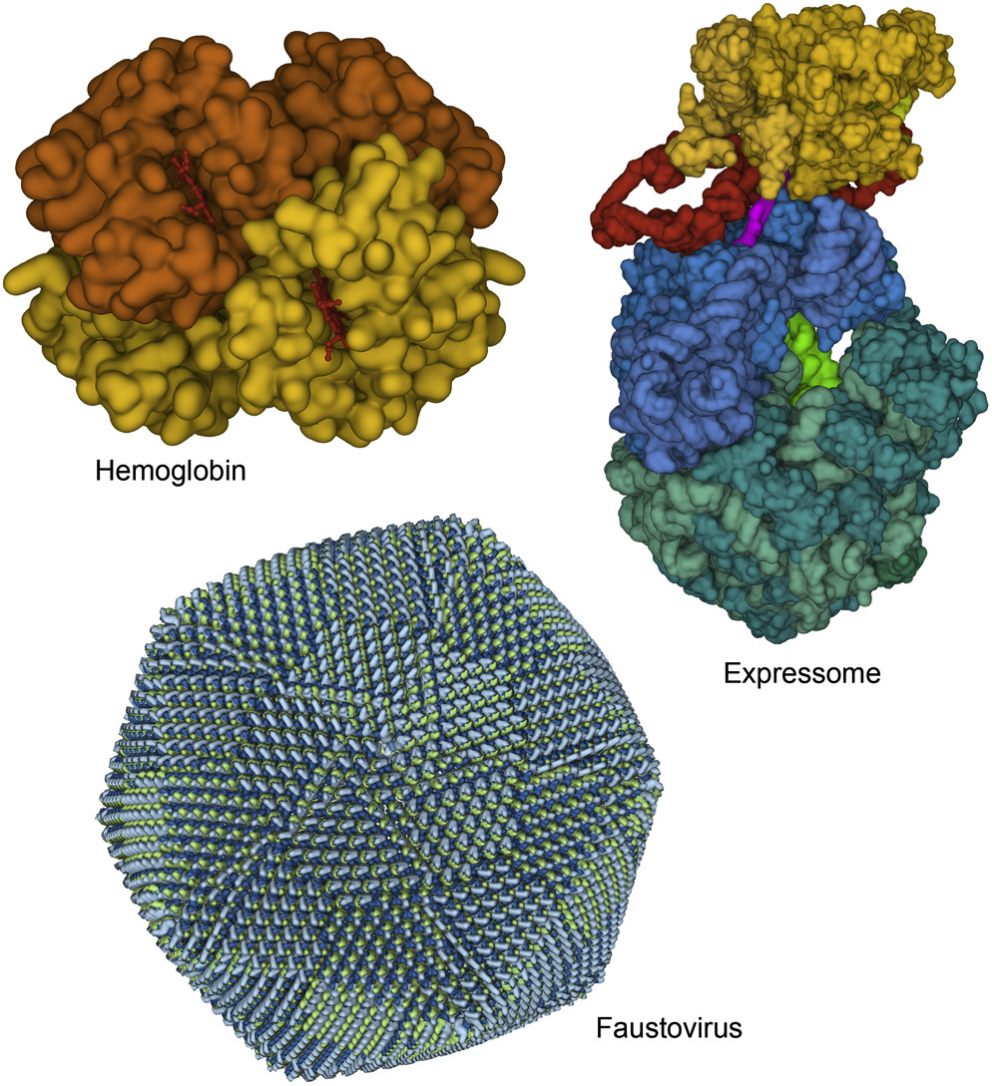
**Gaussian surfaces provide a way to tune a representation based on the complexity of the image.** For hemoglobin (2hhb; (68)), a detailed surface closely follows the contours of atoms. For the expressome (6x9q; (69)), a smoother surface is used to help focus attention on the different functional units in the assembly (RNA polymerase in *yellow*, ribosome in *blue* and *cyan*, tRNA in *green*, transcription factors in *red*, and a nascent mRNA in *magenta*). For faustovirus (5j7v; (70)), one of the largest complexes in the PDB archive, a highly smoothed Gaussian surface is provided by default in Mol∗ to highlight subunits while allowing interactive manipulation. All images created with Mol∗ interactively at the RCSB PDB website.

Structures are getting larger and larger with the current advances in cryoelectron microscopy, and methods are needed for interactive display. A host of simplified surfaces have been conceived and implemented over the years and are increasingly being brought to bear on the problem. For example, Gaussian surfaces approximate the electron density of a molecule by summing Gaussians centered on each atom, then contouring the density. This approach can create representations reminiscent of solvent-accessible surfaces (Fig. 15) and can also be used to create simplified surfaces by tweaking the parameters. The web-based Mol∗ viewer (molstar.org) at the RCSB PDB site currently uses a smooth Gaussian surface as the default for very large structures (Fig. 16). This has two advantages. First, since only the outer surface is seen, it greatly reduces the complexity of the image making the overall structure of the assembly interpretable. Second, the representation is compact enough to allow interactive performance.

### “Seeing” molecules

Once we have chosen a metaphor for the shape and form of the molecule, we then need to turn our attention to metaphors for “seeing.” Given that these images are synthesized digitally, we have unfettered freedom to make choices based on the goals of the image. The field of computer graphics has long been focused on creating photorealistic depictions, so in the first decades of molecular graphics development, a distinctive “shiny plastic” look was widely used, using techniques of Phong shading (49) to generate images reminiscent of the physical space-filling models used by Pauling and later in many laboratories and classrooms. This style of rendering has proven to be quite effective and has been progressively refined with cast shadows and softer ambient occlusion shading, subtler approaches to the shiny specular highlights, depth cueing, and many other rendering tricks of the trade. Together, these generate images that highlight the three-dimensionality of the molecule. The images in Figures 15 and 16 are exemplary of the types of renderings that are provided by default in many contemporary molecular graphics packages using these techniques.

**Figure 17:**
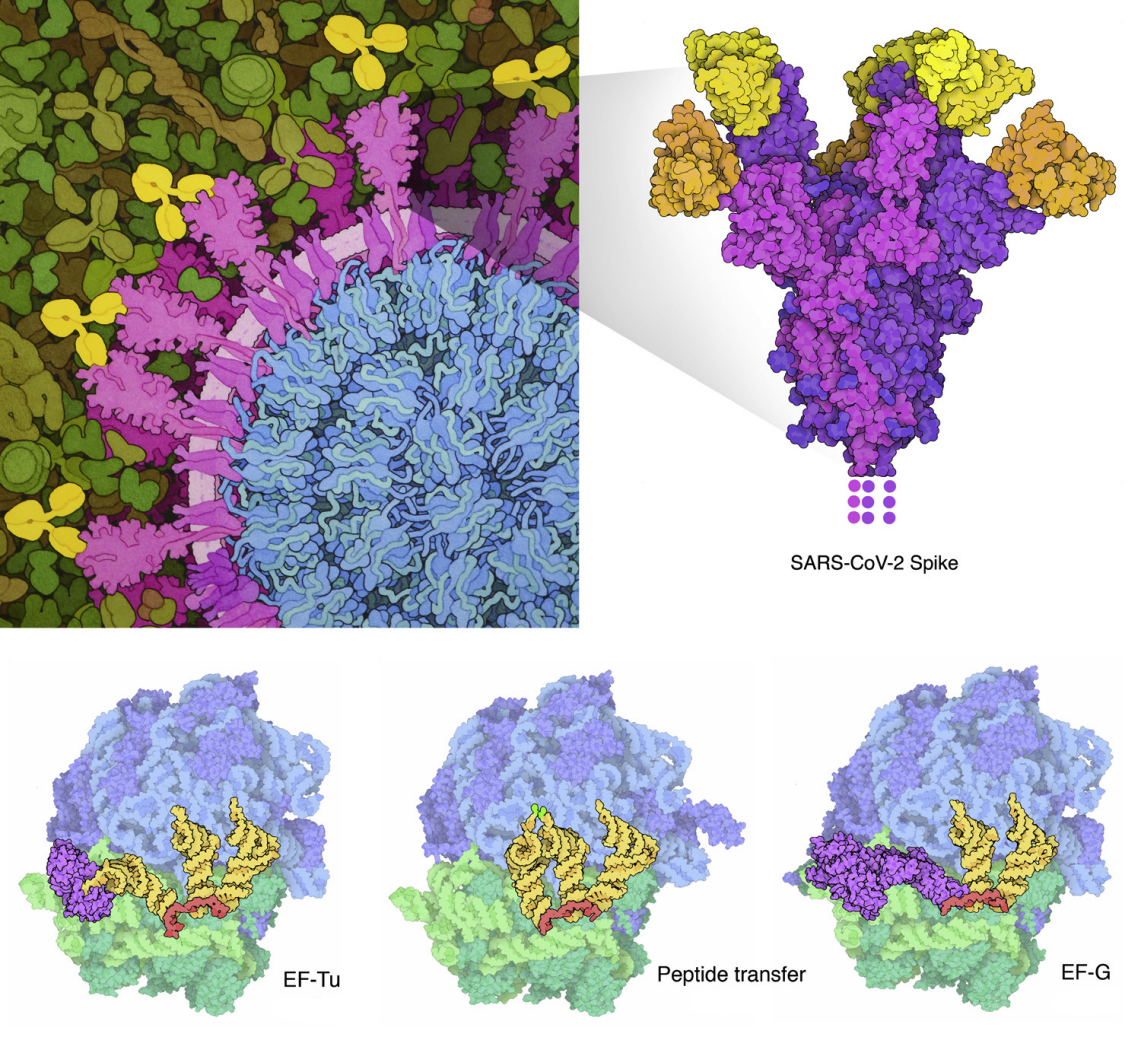
**Nonphotorealistic illustration.***Top*, computer graphics illustration of coronavirus spike protein with bound antibody Fab domains (*right*, 7cwu; (71)) and a hand-drawn artistic conception of this spike-antibody interaction in the context of the whole virus in blood plasma (*left*). *Bottom*, computer graphics illustrations of ribosomes showing three key stages in elongation of protein synthesis (4v5g, 4v5d, 4v5f; (72, 73, 74)). Illustrations are part of outreach materials at PDB-101 (pdb101.rcsb.org).

For many years, we and others have also explored nonphotorealistic approaches to seeing molecules (50), most notably, as part of outreach work with the “Molecule of the Month” feature at PDB-101, the outreach portal of the RCSB Protein Data Bank (51). As perfectly demonstrated in Jane’s hand-drawn diagrams, cartoons are an effective way to simplify a scene, paring it down to the essential components. Our “Illustrate” software generates cartoony images using an imaging processing technique to generate outlines and flat, unshaded atoms to reduce visual clutter (52). The goal of these cartoon illustrations is to give an impression of the subunits in an assembly, rather than focusing on the details of each atom. As with the Gaussian surfaces in Figure 16, they scale nicely in larger scenes using highly simplified cartoons of the molecular shape. For example, I routinely combine digital images of individual molecular structures with paintings of cellular environments that use simpler cartoony shapes for each molecule (Fig. 17). Illustrative images also lend themselves easily to other graphical tricks for telling molecular stories, such as including schematic elements (as in the unobserved membrane-spanning portions of the spike protein) and overlaps and transparency to show processes occurring inside molecular assemblies (as in the three ribosome structures shown here).

### Seeing cellular environments

**Figure 18:**
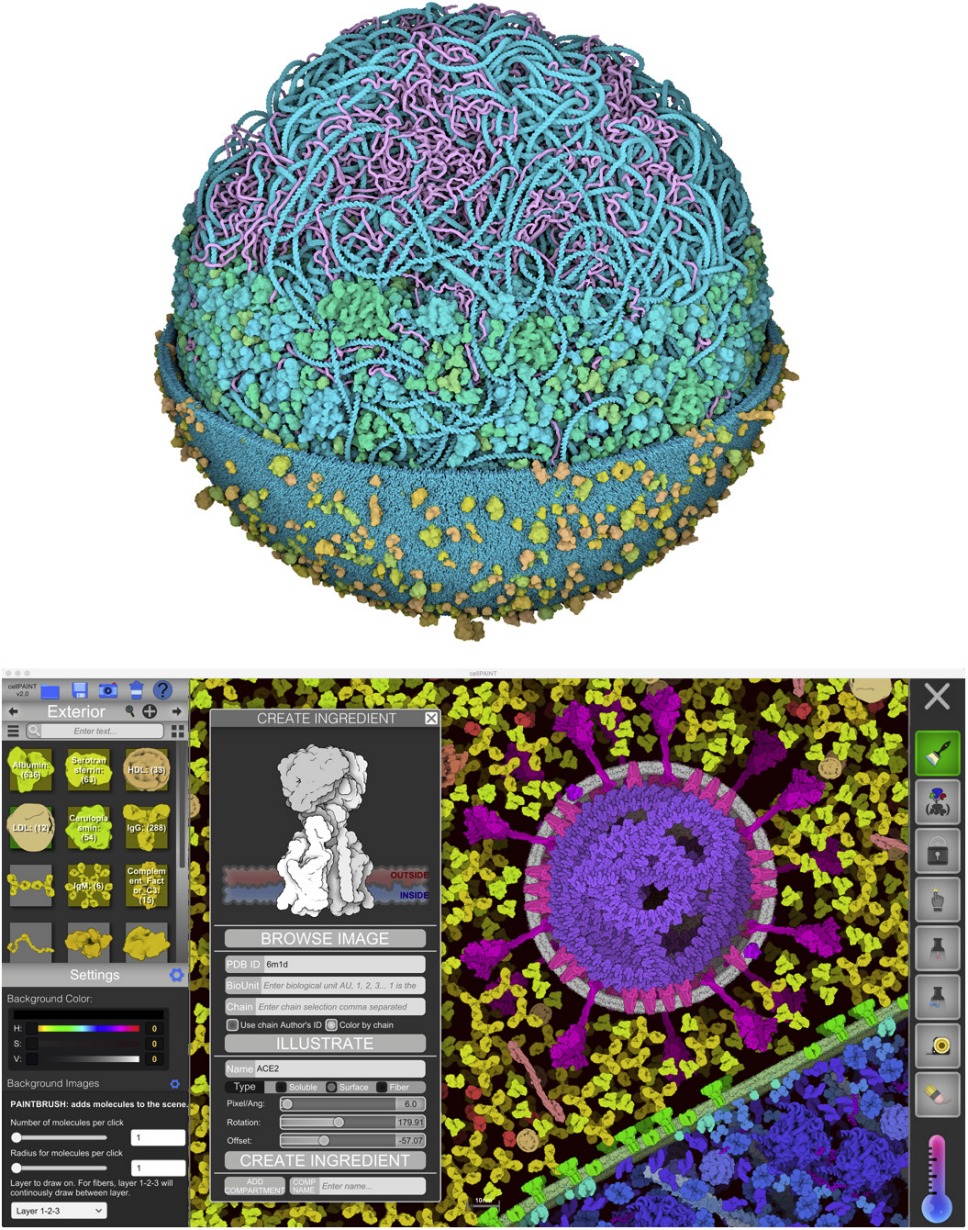
**Modeling and visualization of molecules in cells.***Top*, model of a mycoplasma bacterium based on data from WholeCell models (75). In this computer graphics visualization, the whole model is shown at the base, then in progressive layers, the membrane is removed, and soluble molecules are removed to show only the DNA and RNA. Modeling and visualization by Ludovic Autin and Martina Maritan. *Bottom*, creating an illustration of SARS-CoV-2 using CellPAINT. Molecules are chosen from the palette on the left and interactively painted into the scene. In the pop-up “Create Ingredient” window, the user is reading coordinates for the cellular receptor ACE2 to add to the palette.

As the PDB archive continues to grow, we are rapidly approaching the time when we will have structural snapshots of every molecule in a cell. Combined with the ever-finer resolution of cryoelectron tomography, it is becoming feasible to create integrative models of entire cells at molecular detail. This goal poses several grand challenges, including the gathering and curation of data from multiple sources, the complexity of the higher-order assemblies and ultrastructure of cells, and the limited (but constantly improving) performance of available hardware, all of which are being addressed by multiple laboratories around the world (53). We are taking two approaches, designed for two different but complementary goals, and leveraging the powerful advances in graphics software from the gaming community (Fig. 18). The first is CellPACK, a suite of programs designed to create models of cellular environments based on a recipe of all the component molecules and where they reside in the cell (54). The second is CellPAINT, an interactive program much like a digital painting program that allows nonexpert users to build illustrations of cellular environments (55). This type of guided image creation is designed to give users some of the freedom that is possible in hand-drawn images, providing tools that enforce known relative sizes and behaviors of molecules while allowing easy, interactive construction of diverse subcellular scenes comprising these molecules.

### New ways of seeing molecules for the challenges of today

The molecular graphics techniques in widespread use today—bond diagrams, ribbon diagrams, dot surfaces, and space-filling representations—are highly effective for research, dissemination, and education/outreach (43, 56, 57, 58, 59). However, the field of structural biology continues to expand, and the classic graphical metaphors are providing the seeds for further exploration and development. To me, the most exciting aspect of molecular graphics is that there is always a new challenge. Here are a few selected examples of avenues that are calling for innovation in the near future and a few of the ways they are being addressed by the creative molecular graphics community.

The dynamic nature of biomolecules continues to be a central challenge. This is particularly problematic, and interesting, in the growing field of intrinsically disordered proteins, which undergo large structural transitions in their functional roles, or perhaps never adopt a defined structure at all. Dynamics is also an intrinsic part of nearly every biomolecular process, from the allosteric motions of hemoglobin to the many complex steps of ribosomal initiation, elongation, and termination. Our visual tools for exploring and disseminating this dynamic information are woefully limited. A snapshot metaphor is often used for defined dynamic processes such as allosteric transitions and pathways of enzyme catalysis. In publication, these snapshots are often captured in a series of small multiple images. Kinemages (21), JSmols (60), and the like add personal agency to these snapshots, allowing users to flip between conformations and interactively explore various aspects of the structures. Animation also remains a primary tool for representing dynamics, borrowing metaphors from cinema to create molecular stories that capture salient dynamic events (61). These are necessarily scripted, since biomolecular processes typically occur over large numbers of time steps and key transitions are often lost in a sea of random motion. This requires much effort and artistry to focus attention and employs many approximations to create a comprehensible presentation of digestible length.

For decades, molecular graphics researchers have explored the newest technology to improve the user experience when interacting with molecules. As described above, molecular graphics was one of the driving applications for the first interactive computer graphics hardware and provided an ongoing test-bed for development of virtual reality applications. New metaphors were developed to streamline the user experience in these applications, as they require both comprehensible representations of the molecules (that are compact enough for interactive performance) and intuitive methods for navigating the space around them. All manner of grabbing, turning, walking, and teleporting metaphors have been tried, within spaces that represent a “Fantastic Voyage,” molecular museum, virtual laboratory, and many other imaginative scenarios (see (62) and the many projects cited there). Given the growing widespread availability of immersive headsets, and also interactive touch-sensitive tablets, we expect that there is a rich environment for continued experimentation. Currently, we also have the great advantage of being able to leverage the massive development effort that is being applied to the gaming industry, providing turnkey development environments such as Unity for creating our molecule-based applications.

We are also seeing molecular graphics researchers going back to their roots and turning once again to physical models as a way to provide tangible understanding of biomolecular structure and function. The growing availability of 3D printing technology has opened the door to creating tangible models based on atomic coordinate files (63, 64). This has posed new challenges for molecular metaphors, since an orthogonal set of design constraints are required on top of the typical visual design constraints. For printed models, the representation must be strong enough to withstand printing and handling, often requiring the addition of support struts and other elements related to the physical structure. These elements must be carefully designed so as to not distract from understanding of the underlying biology.

Perhaps the most exciting short-term challenge facing the molecular graphics community is the current revolution in cryoelectron microscopy (34). Cryo-EM is revealing the structures of biomolecular assemblies of unprecedented size and complexity and is consequently breaking many of the familiar methods that we use for exploring molecular structure. New methods are needed to make these complex structures accessible, easily and intuitively (65). A multiresolution approach will undoubtedly be necessary, allowing users to flip between detailed exploration of protein folds and sidechain interactions and larger-scale understanding of the assembly of subunits and comparison with other assemblies. This will pose great challenges in the design of effective representations at all of the levels, methods for facile navigation between these levels, and implementation of these methods in infrastructure powerful enough to provide interactive performance. Solutions to these issues are already well under way in projects like ChimeraX (38) and Mol∗, promising exciting new developments to come.

## Conflict of interest

The authors declare that they have no conflicts of interest with the contents of this article.
